# Impact of diabetes mellitus on disease severity and patient survival in idiopathic pulmonary arterial hypertension: data from the Polish multicentre registry (BNP-PL)

**DOI:** 10.1186/s12933-023-01885-6

**Published:** 2023-07-13

**Authors:** Kamil Jonas, Marcin Kurzyna, Ewa Mroczek, Łukasz Chrzanowski, Tatiana Mularek-Kubzdela, Ilona Skoczylas, Piotr Błaszczak, Grzegorz Grześk, Katarzyna Mizia-Stec, Beata Kuśmierczyk, Karol Kamiński, Ewa Lewicka, Małgorzata Peregud-Pogorzelska, Michał Tomaszewski, Wojciech Jacheć, Zbigniew Gąsior, Agnieszka Pawlak, Robert Ryczek, Piotr Pruszczyk, Anna Doboszyńska, Katarzyna Widejko-Pietkiewicz, Wiesława Zabłocka, Marcin Waligóra, Grzegorz Kopeć

**Affiliations:** 1 Department of Cardiac and Vascular Diseases, John Paul II Hospital in Krakow, Krakow, 31-202 Poland; 2 Pulmonary Circulation Centre, Department of Cardiac and Vascular Diseases, Faculty of Medicine, Jagiellonian University Medical College, Krakow, 31-008 Poland; 3 Center for Innovative Medical Education, Department of Medical Education, Faculty of Medicine, Jagiellonian University Medical College, Krakow, 30-688 Poland; 4 Department of Pulmonary Circulation, Thromboembolic Diseases and Cardiology, Centre of Postgraduate Medical Education, Fryderyk Chopin Hospital in European Health Centre Otwock, Otwock, Poland; 5 Clinic of Heart Diseases, Institute of Heart Diseases, University Clinical Hospital, Wrocław, Poland; 6 Cardiology Department, Medical University of Lodz, Lodz, 91-347 Poland; 7 Department of Cardiology, Poznan University of Medical Sciences, Poznan, 61-701 Poland; 8 3rd Department of Cardiology, Faculty of Medical Sciences in Zabrze, Medical University of Silesia, Katowice, 41-800 Poland; 9 Department of Cardiology, Cardinal Wyszynski Hospital, Lublin, 20-718 Poland; 10 Department of Cardiology and Clinical Pharmacology, Collegium Medicum in Bydgoszcz, Nicolaus Copernicus University in Toruń, Toruń, Poland; 11 First Department of Cardiology, School of Medicine in Katowice, Medical University of Silesia in Katowice, Katowice, 40-635 Poland; 12 Department of Congenital Heart Disease Institute of Cardiology, Warsaw, 04-628 Poland; 13 Department of Cardiology, Medical University of Bialystok, Bialystok, 15-276 Poland; 14 Department of Population Medicine and Civilization Diseases Prevention, Medical University of Bialystok, Bialystok, 15-269 Poland; 15 Department of Cardiology and Electrotherapy, Medical University of Gdansk, Gdansk, 80-211 Poland; 16 Department of Cardiology, Pomeranian Medical University, Szczecin, 70-111 Poland; 17 Department of Cardiology, Medical University of Lublin, Lublin, 20-090 Poland; 18 2nd Department of Cardiology, School of Medicine with Dentistry Division in Zabrze, Medical University of Silesia in Katowice, Zabrze, 41-800 Poland; 19 Department of Cardiology, School of Health Sciences, Medical University of Cardiology in Katowice, Katowice, 40-635 Poland; 20 Department of Invasive Cardiology, Polish Academy of Sciences, Mossakowski Medical Research Centre, Central Clinical Hospital of the Ministry of Interior, Warsaw, 02-507 Poland; 21 Department of Cardiology and Internal Medicine, Military Institute of Medicine - National Research Institute, Warsaw, 04-141 Poland; 22 Department of Internal Medicine and Cardiology with the Center for Diagnosis and Treatment of Venous Thromboembolism, Medical University of Warsaw, Warszawa, Poland; 23 Pulmonary Department, University of Warmia and Mazury, Olsztyn, 10-357 Poland; 24 Department of Cardiology, Copper Health Center, Lubin, 59-300 Poland; 25 Department of Cardiology, Provincial Specialist Hospital in Szczecin, Szczecin, Poland

**Keywords:** Diabetes, Pulmonary arterial hypertension, Mortality, Glucose metabolism

## Abstract

**Background:**

Recent studies revealed that alterations in glucose and lipid metabolism in idiopathic pulmonary arterial hypertension (IPAH) are associated with disease severity and poor survival. However, data regarding the impact of diabetes mellitus (DM) on the prognosis of patients with IPAH remain scarce. The aim of our study was to determine that impact using data from a national multicentre prospective pulmonary hypertension registry.

**Methods:**

We analysed data of adult patients with IPAH from the Database of Pulmonary Hypertension in the Polish population (BNP‑PL) between March 1, 2018 and August 31, 2020. Upon admission, clinical, echocardiographic, and haemodynamic data were collected at 21 Polish IPAH reference centres. The all-cause mortality was assessed during a 30-month follow-up period. To adjust for differences in age, body mass index (BMI), and comorbidities between patients with and without DM, a 2-group propensity score matching was performed using a 1:1 pairing algorithm.

**Results:**

A total of 532 patients with IPAH were included in the study and 25.6% were diagnosed with DM. Further matched analysis was performed in 136 patients with DM and 136 without DM. DM was associated with older age, higher BMI, more advanced exertional dyspnea, increased levels of N-terminal pro–brain natriuretic peptide, larger right atrial area, increased mean right atrial pressure, mean pulmonary artery pressure, pulmonary vascular resistance, and all-cause mortality compared with no DM.

**Conclusions:**

Patients with IPAH and DM present with more advanced pulmonary vascular disease and worse survival than counterparts without DM independently of age, BMI, and cardiovascular comorbidities.

## Background

Pulmonary arterial hypertension (PAH) is a rare multifactorial disease characterized by an increase in pulmonary vascular cell proliferation, vasoconstriction of small pulmonary arteries, and increase in pulmonary vascular resistance (PVR) [1, 2]. Remodeling of the pulmonary vasculature leads to progressive symptoms of heart failure and an increased risk of mortality. Recent studies of our and other research groups have revealed that patients with idiopathic PAH (IPAH) have elevated blood glucose levels, an increased prevalence of diabetes mellitus (DM), and an altered lipid profile compared to the general population [3–7]. Moreover, metabolic parameters were correlated with disease severity and were able to predict poor survival in this group. Importantly, DM was recently identified as an cardiopulmonary comorbidity associated with a new phenotype of IPAH with a diminished response to PAH medication [1].

Further investigations demonstrated that patients with IPAH more often had glucose intolerance and decreased insulin secretion in response to the oral glucose tolerance test compared to matched controls [8]. These findings were independent of age and body mass index (BMI) and were associated with circulating inflammatory markers and reduced exercise capacity [8]. In a study using the hyperglycemic clamp technique and metabolomic analysis in a small subset of patients with PAH, the authors confirmed that inadequate glucose control in IPAH may, in fact, result from elevated hepatic insulin extraction and a shift in metabolism favoring lipids and ketones [9]. Notably, a significant role of insulin resistance in the progression of pulmonary vascular disease has been previously described in an animal model and in patients with PAH [10, 11]; however, data regarding the impact of DM on the prognosis of patients with IPAH still remain scarce. Therefore, the aim of our present study was to determine the impact of DM on pulmonary hypertension severity and prognosis in patients with IPAH using data from a national multicentre prospective pulmonary hypertension registry.

## Methods

### Study population

The study included data from the Database of Pulmonary Hypertension in the Polish population (BNP‑PL) of adult patients with IPAH diagnosed or treated between March 1, 2018, and August 31, 2020, which has been described previously [12, 13]. They were followed until August 31, 2021. Upon admission, clinical, echocardiographic, and haemodynamic data were collected at 21 Polish PH reference centres accredited by the National Health Fund.

The diagnosis of IPAH was established by confirming precapillary pulmonary hypertension (elevated mean pulmonary artery pressure [mPAP] ≥ 25 mmHg and pulmonary artery wedge pressure [PAWP] ≤ 15 mmHg) with elevated PVR (> 3 Wood units [WU]). Patients with findings typical for chronic thromboembolic pulmonary hypertension, significant lung disease, or with other known causes of PAH were classified as having other types of pulmonary hypertension according to the European Society of Cardiology (ESC) guidelines, and thus were not included in the study. The follow-up data regarding morbidity and mortality were collected during follow-up visits and telephone contacts by the participating centres. We identified cardiovascular comorbidities based on the patients’ previous medical history and diagnostic tests performed during the initial diagnosis of PAH as needed. Patients were assessed for DM at baseline. The baseline assessment included data acquired at the time of PAH diagnosis in incident cases (diagnosed after the beginning of the BNP-PL registry, which was on March 1, 2018) and data obtained at the most recent visit in previously treated patients (diagnosed before March 1, 2018). The date of diagnosis was defined as the date of the first right heart catheterization that met the hemodynamic criteria for pre-capillary pulmonary hypertension [12]. We defined patients with DM if any of the following was true: previous diagnosis of any type of diabetes, on treatment approved for DM, fasting blood glucose levels ≥ 126 mg/dL (7.0 mmol/L) documented on 2 different days, or blood glucose levels ≥ 200 mg/dL (11.1 mmol/L) at the 120-minute time point of the oral glucose tolerance test. Patients who met none of these criteria were deemed not to have DM. Coronary artery disease was diagnosed based on current or previous coronary angiography or evidence of myocardial ischemia. We actively searched for coronary artery disease in symptomatic patients according to the ESC guidelines.The diagnosis of chronic kidney disease was based on documented GFR < 60ml/min/1.73 m² in the patient’s medical history or during the initial diagnostic workup for PAH. The severity of IPAH was assessed at baseline using parameters recommended by the ESC [2]: heart failure symptoms, World Health Organization functional class (WHO-FC), serum N-terminal pro–brain natriuretic peptide (NT-proBNP) levels, the 6-minute walk distance (6MWD) test, echocardiography parameters including assessment of pericardial effusion and right atrial area, haemodynamic parameters including mPAP, mixed venous oxygen saturation (SvO_2_), right atrial pressure (RAP), PVR, and cardiac index (CI). All patients provided written informed consent prior to entering the study. The study protocol conforms to the ethical guidelines of the Declaration of Helsinki and was revised and approved by the local ethics committee.

### Statistical analysis

**Fig. 1:**
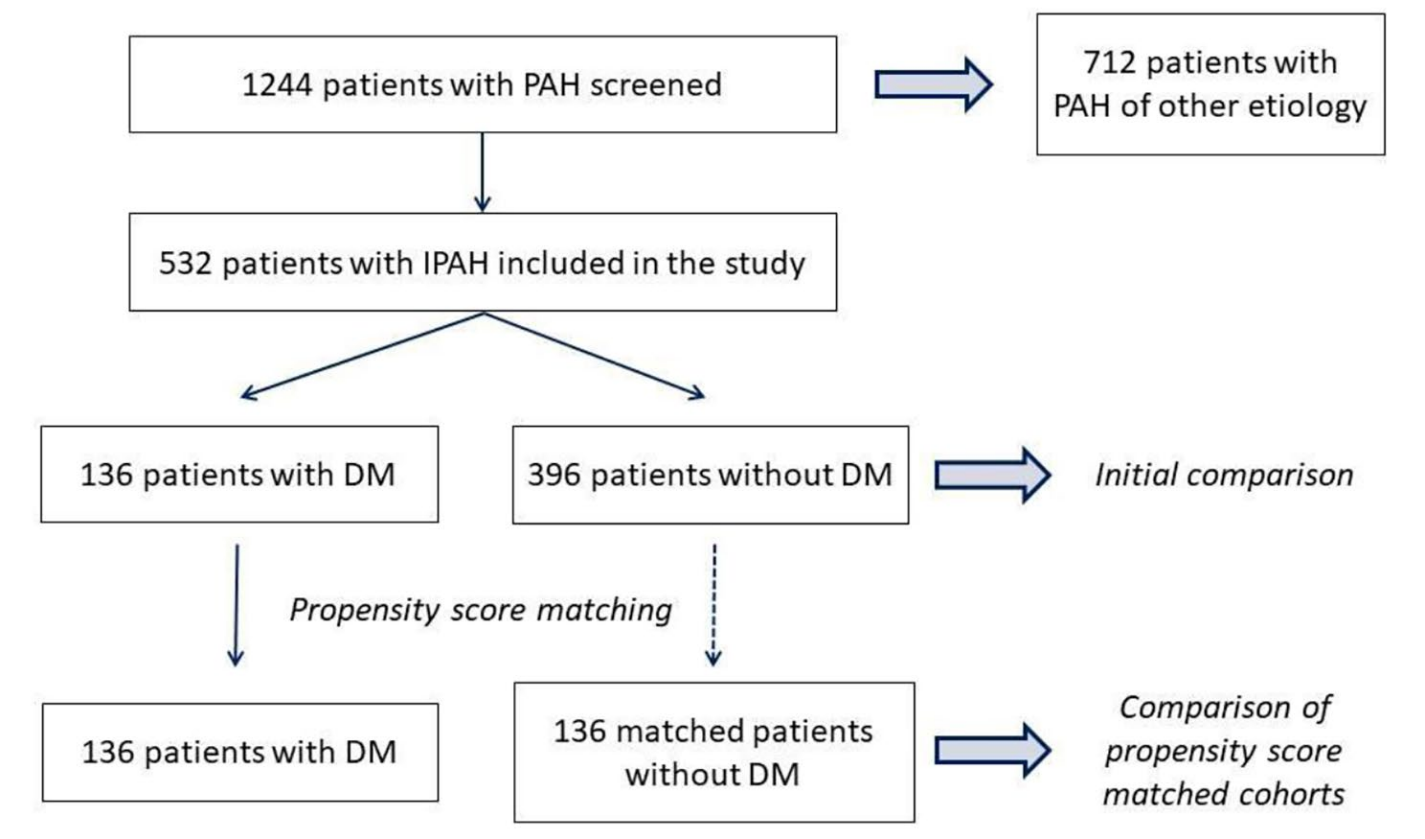
Flowchart representing details of the patient recruitment. DM diabetes mellitus, PAH pulmonary artery hypertension

Continuous variables are presented as median (interquartile range) and categorical variables as count (percentage). Categorical variables were compared using the chi-square test. Continuous variables were compared between groups using the Mann-Whitney U test, according to data distribution. Firstly, we assessed the entire population at baseline. To control for the inherent differences between patients with IPAH with and without DM that might act as confounding factors, we established a propensity score for the main variables found to differ significantly (*P* < 0.05) according to DM status. Matching was performed using the following variables: age, obesity, history of coronary artery disease, arterial hypertension, and chronic renal failure (Table 1). Matching was performed with the nearest neighbour method, using a 1:1 pairing with replacement. The propensity score model was verified by comparing covariate distribution between the 2 patient groups. Further analysis was performed in matched cohorts at baseline and during follow-up to assess all-cause mortality. The Kaplan–Meier method with the chi-square test was used for survival analysis. To further assess the relationship between DM and the risk of all-cause mortality, we used Cox proportional hazards regression. Models were hierarchically adjusted for major cardiovascular comorbidities and markers of PAH severity. Details of the patient recruitment are presented in the study flow chart (Fig. 1). The level of statistical significance was set at 0.05. Statistical analysis was performed with the use of the Dell Statistica data analysis software system version 13.3 (TIBCO Software Inc., Palo Alto, California, United States) and MedCalc version 19.2.6 (MedCalc Software, Ostend, Belgium).

**Table 1:** Baseline characteristics of patients with idiopathic pulmonary arterial hypertension according to diabetes status

	Unmatched cohort			Matched cohort		
	No DM n = 396	DM n = 136 (25.6%)	p value	No DM n = 136	DM n = 136	p value
Age (years)	56.4 [40.7–67.9]	70.4 [64.6–76.0]	< 0.001*	70.3 [64.1–74.5]	70.4 [64.6–76.0]	0.7
Female sex	281 (71.0%)	85 (62.5%)	0.07	94 (69.1%)	85 (62.5%)	0.3
BMI	25.7 [22.3–29.7]	29.7 [26.4–33.3]	< 0.001*	29.7 [25.8–32.6]	29.7 [26.4–33.3]	0.6
Arterial Hypertension	161 (40.7%)	117 (86.0%)	< 0.001*	118 (86.8%)	117 (86.0%)	0.9
Current smoking	17 (4.3%)	9 (6.6%)	0.3	5 (3.7%)	9 (6.6%)	0.3
Coronary artery disease	60 (15.2%)	49 (36.0%)	< 0.001*	46 (33.8%)	49 (36.0%)	0.7
COPD/Asthma	59 (14.9%)	30 (22.0%)	0.06	21 (15.4%)	30 (22.0%)	0.1
Chronic renal failure	47 (11.9%)	50 (36.8%)	< 0.001*	48 (35.3%)	50 (36.8%)	0.8
Beta-blocker	134 (33.8%)	87 (64.0%)	< 0.001*	74 (54.4%)	87 (64.0%)	0.1
Statins	120 (30.3%)	93 (68.4%)	< 0.001*	91 (66.9%)	93 (68.4%)	0.8
Anticoagulants	116 (29.3%)	60 (44.1%)	0.002*	77 (56.6%)	60 (44.1%)	0.06
ACEI/ARB	95 (23.9%)	74 (54.4%)	< 0.001*	65 (47.8%)	74 (54.4%)	0.3
Loop diuretics	248 (62.6%)	123 (90.4%)	< 0.001*	96 (70.6%)	123 (90.4%)	< 0.001*
ASA	50 (12.6%)	47 (34.6%)	< 0.001*	31 (22.8%)	47 (34.6%)	0.03*
Metformin	-	70 (51.5%)	-	-	-	-
Insulin	-	35 (25.7%)	-	-	-	-
Sulfonylureas	-	41 (30.1%)	-	-	-	-
Acarbose	-	7 (5.1%)	-	-	-	-
DPP-4 inhibitors	-	2 (1.4%)	-	-	-	-
SGLT-2 inhibitors	-	2 (1.4%)	-	-	-	-
PAH-specific treatment	391 (98.7%)	136 (100%)	0.2	135 (99.3%)	136 (100%)	0.3
PAH combination therapy	267 (67.4%)	101 (74.3%)	0.1	89 (65.4%)	101 (74.3%)	0.1
Parenteral prostacyclin	114 (28.8%)	35 (25.7%)	0.5	31 (22.8%)	35 (25.7%)	0.6

Data are presented as median (interquartile range) or number (percentage). Asterisk indicates statistically significant values

*ACEI* angiotensin-converting enzyme inhibitors, *ARB* angiotensin receptor blockers, *ASA* acetylsalicylic acid, *BMI* body mass index, *COPD* chronic obstructive pulmonary disease, *DM* diabetes mellitus, *PAH* pulmonary arterial hypertension

## Results

### Study population

The study included a total of 532 patients with IPAH, 136 of whom (25.6%) were diagnosed with DM. Further analysis of matched cohorts was performed in 272 patients: 136 with DM and 136 without DM. Baseline characteristics of recruited patients are presented in Table 1. We found that in the unmatched population, DM was associated with a distinct clinical phenotype, including older age, higher BMI, and higher frequency of other cardiovascular comorbidities, including arterial hypertension, coronary artery disease, and chronic kidney disease. Cardiovascular medications, such as beta-blockers, statins, angiotensin-converting enzyme inhibitors/angiotensin receptor blockers, loop diuretics, and acetylsalicylic acid, were more frequently administered to patients with DM than to other participants. The between-group differences in the use of acetylsalicylic acid and loop diuretics remained significant after matching. There were no differences between the groups, both before and after matching, in the use of PAH-specific treatment, including PAH combination therapy and parenteral prostanoids. Due to the design of the BNP-PL database, all variables included in Table 1 were 100% complete as they were mandatory for record creation.

### Clinical assessment

Markers of pulmonary hypertension severity in patients with and without DM are presented in Table 2. In the IPAH population, advanced exertional dyspnea defined as WHO-FC class III or IV was present in 75% of patients with DM and in 44% of those without DM. Similar distribution was also found in the matched cohorts (75% vs. 55% of patients, respectively, p < 0.001). Only 25% of patients with DM presented with mild or without dyspnea on exertion (WHO-FC class I or II). Patients with DM had poorer exercise tolerance (as measured by the 6MWD test) compared with the subjects without DM both before (301 vs. 423 m; p < 0.001) and after matching (301 vs. 335 m; p = 0.03). Patients with DM also had significantly higher plasma NT-proBNP levels (1628 vs. 461 pg/ml; p < 0.001 and 1628 vs. 719 pg/ml; p = 0.003, respectively) and a larger right atrial area as assessed by echocardiography (26 vs. 22 cm^2^, p < 0.001 and 26 vs. 24 cm^2^, p = 0.04, respectively) than patients without DM.

**Table 2:** Severity of pulmonary hypertension according to diabetes status

	Unmatched cohort			Matched cohort		
	No DM n = 396	DM n = 136	p value	No DM n = 136	DM n = 136	p value
**Clinical parameters**						
WHO-FC N = 530 (99.6%)						
I II III IV	29 (7.3%) 191 (48.5%) 155 (39.3%) 19 (4.8%)	1 (0.7%) 33 (24.4%) 77 (57.0%) 24 (17.8%)	< 0.001*	1 (0.7%) 60 (44.1%) 67 (49.2%) 8 (5.9%)	1 (0.7%) 33 (24.4%) 77 (57.0%) 24 (17.8%)	< 0.001*
NT-proBNP (pg/ml) N = 500 (94.0%)	461 [130–1812]	1628 [470–3058]	< 0.001*	719 [240–2187]	1628 [470–3058]	0.003*
6MWD test (m) N = 491 (92.3%)	423 [307–504]	301 [177–378]	< 0.001*	335 [220–441]	301 [177–378]	0.03*
**Echocardiography**						
Pericardial effusion N = 508 (95.5%)	69 (18%)	25 (19%)	0.8	22 (16.8%)	25 (19%)	0.6
RAA (cm^2^) N = 487 (91.5%)	22 [18–28]	26 [21.5–32.0]	< 0.001*	24 [19–28.9]	26 [21.5–32.0]	0.04*
TAPSE (mm) N = 458 (86.1%)	19 [16–22]	19 [16–22]	0.7	19 [16–22]	19 [16–22]	0.8
**Right heart catheterization**						
mPAP (mmHg) N = 521 (97.9%)	44.5 [35–55]	46 [36–55]	0.5	40 [33–51]	46 [36–55]	0.01*
PAWP (mmHg) N = 480 (90.2%)	9 [6–12]	9 [7–13]	0.06	10 [7–12]	9 [7–13]	0.9
SvO_2_ (%) N = 488 (91.7%)	69 [64–75]	65 [58–71]	< 0.001*	66 [60–71]	65 [58–71]	0.4
PVR (WU) N = 513 (96.4%)	7.6 [5.1–11.0]	7.8 [5.2–11]	0.8	6.3 [5–8.7]	7.8 [5.2–11]	0.02*
CI (l/min/m^2^) N = 505 (94.9%)	2.6 [2.1–3.2]	2.4 [2.0–2.8]	0.06	2.6 [2.1–2.8]	2.4 [2.0–2.8]	0.3
RAP (mmHg) N = 505 (94.9%)	6 [4–9]	8 [5–12]	< 0.001*	7 [3–9]	8 [5–12]	0.003*

Asterisk indicates statistically significant values, N (%) indicates the number of patients’ data available for each variable analyzed

*CI* cardiac index, *DM* diabetes mellitus, *mPAP* mean pulmonary artery pressure, *NT-proBNP* N-terminal pro–brain natriuretic peptide, *PAWP* pulmonary artery wedge pressure, *PVR* pulmonary vascular resistance, *RAA* right atrial area, *RAP* right atrial pressure, *6MWD* 6-minute walk distance, *SvO*_*2*_ mixed venous oxygen saturation, *TAPSE* tricuspid Annular Plane Systolic Excursion, *WHO-FC* World Health Organization functional class

### Right heart catheterization

Assessment of the hemodynamic profile showed a significantly higher mean right atrial pressure in patients with DM than in the population without DM both before and after matching (8 vs. 6 mmHg; p < 0.001 and 8 vs. 7 mmHg; p = 0.003, respectively). Patients with DM also had higher mPAP (46 vs. 40 mmHg; p = 0.01) and PVR (7.8 vs. 6.3 WU; p = 0.02) than individuals without DM. Importantly, no differences in PAWP were found between the groups.

### All-cause mortality

**Fig. 2:**
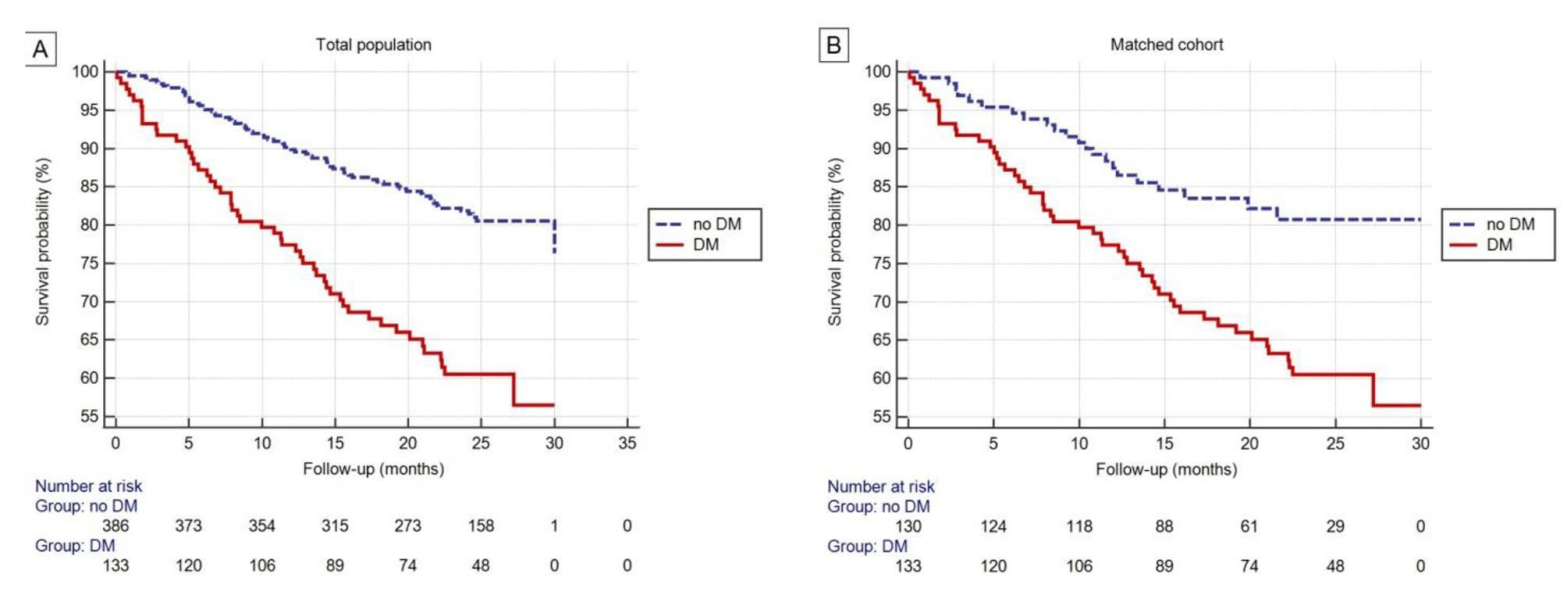
All-cause mortality. Kaplan–Meier survival curves for all-cause mortality according to diabetes status in the whole population (A; p < 0.001) and in the matched cohort (B; p = 0.01) DM diabetes mellitus

During the mean observation period of 21 months, we recorded a total of 122 deaths, which accounted for 22.9% of the study population. All-cause mortality was higher in patients with DM than in patients without DM. After matching, DM status remained the only factor impacting all-cause mortality (p < 0.001). Details of survival analysis using the Kaplan–Meier method are shown in Fig. 2. Using Cox proportional hazards regression, we found that DM was significantly associated with all-cause mortality in unadjusted analysis (HR: 2.46, 95% CI: 1.71 to 3.53, p < 0.0001) and after hierarchical adjustment for known and potential risk factors for mortality in PAH. The fully-adjusted model, which included DM, age, sex, BMI, coronary artery disease, COPD/asthma, hypertension, chronic renal failure, WHO-FC, 6MWD, NT-proBNP, RAA, TAPSE, CI, mPAP, PVR, RAP, and SvO2, showed that significant predictors of death were: DM and age (HR: 1.03; 95% CI: 1.01–1.05), 6MWD (HR: 0.997; 95% CI: 0.994–0.998), and RAP (HR: 1.08; 95% CI: 1.03–1.14).

## Discussion

The main finding of our study is that patients with IPAH and DM have more advanced pulmonary vascular disease and worse survival than those without DM. Importantly, these findings are independent of age, BMI, and associated cardiovascular comorbidities and treatments. This is the first report from a large, multicentre registry describing the impact of DM on long-term survival in a selected population of patients with IPAH.

Detrimental effect of DM on left ventricular dysfunction, systemic vascular remodeling, and long-term prognosis has been previously reported in multiple populations including patients with coronary artery disease [14, 15], heart failure [16, 17], or hypertrophic cardiomyopathy [18]. Endothelial dysfunction, vasoconstriction, and atherosclerosis were proposed as potential mechanisms of these associations. A similar detrimental effect of DM and altered glucose homeostasis, with a possible distinct mechanism, has also been recently noted in the pulmonary circulation with regard to right ventricular function and pulmonary vascular remodeling [19, 20].

As previously described in a single-centre study of 113 patients by Benson et al. [19], DM in patients with PAH might have an impact on their long-term prognosis as it influences the function of the right ventricle independently of traditional cardiovascular risk factors, including weight and age. However, the conclusions were limited due to the size of the study group and number of events [19].

In another report, Abernethy et al. [21] demonstrated that DM had a detrimental effect on survival in patients with different groups of pulmonary hypertension. Patients with DM were also more likely to present with postcapillary disease as determined by higher levels of PAWP and were less responsive to acute vasoreactivity testing. They also had higher levels of RAP measured during right heart catheterization.

This corroborates our observations in the IPAH population. DM was unequivocally associated with higher levels of RAP and more frequent use of loop diuretics indicating more advanced right ventricular failure. Moreover, in our study, patients with IPAH and DM presented with higher levels of mPAP and PVR, suggesting more advanced pulmonary vascular disease. This might indicate that mechanisms leading to increased mortality in the DM group might be directly associated with promoting pulmonary vascular remodeling, as was previously described in the experimental models of PAH [10, 11].

A series of studies by Heresi et al. [8] provided further insight into the complex glucose haemostasis in PAH. They found that patients with IPAH had impaired glucose tolerance and reduced glucose-stimulated insulin secretion compared with matched controls. This was also associated with increased levels of circulating inflammatory markers. They also showed that insulin response to hyperglycaemia, as assessed by the comprehensive hyperglycaemic clamp technique, was decreased in patients with PAH after matching for age, BMI, and sex. This was attributed to the domination of lipid and ketone metabolism at the expense of glucose control [9]. Similarly, other studies identified elevated levels of haemoglobin A_1c_, glucose intolerance, and pancreatic β-cell dysfunction in patients with IPAH [8]. In our previous studies, we have also found changes in glucose metabolism in patients with pulmonary hypertension. DM and elevated fasting glucose levels were more common in patients with IPAH when compared with individuals without IPAH [7]. Furthermore, in our study, we showed that elevated fasting glucose levels were also associated with a worse prognosis, independently of known markers of disease severity.

Large ongoing registries, including a French registry [22], COMPERA [23], REVEAL [24], SPAHR [25], and many others, have allowed the creation of multicomponent risk stratification tools for patients with PAH that include clinical, imaging, laboratory, and haemodynamic data. In recent years, impaired glucose and insulin metabolism were proposed as novel risk factor in the pathobiology of PAH [4]. A growing body of evidence suggests that impaired glucose homeostasis and DM contribute significantly to the progression of pulmonary vascular disease. This is especially interesting, taking into consideration the potential availability of medications modifying glucose metabolism able to reverse pulmonary vascular remodeling in vitro and in animal models. It also remains to be established whether these patients would benefit from treatment of insulin resistance or better control of DM early in the clinical course.

The exact mechanism by which glucose metabolism affects pulmonary circulation in not fully understood; however, it has been investigated in previous experimental studies. Numerous metabolic alterations, such as insulin resistance, low plasma adiponectin levels, and reduced expression of apolipoprotein E, have been proposed to induce PAH in an animal model [26]. It is also believed that chronic inflammation and altered immunity play an important pathophysiological role in the regulation of glucose homeostasis. In experimental studies, an upregulated inflammatory response, including elevated levels of interleukin (IL) 1β, IL-2, IL-6, IL-8, IL-10, tumour necrosis factor α, and monocyte chemoattractant protein 1, has been shown to precede vascular remodeling and was associated with altered vascular cell metabolism and consequent proliferation of pulmonary artery smooth muscle cells [27–31]. Another study assessing the proliferation of pulmonary artery smooth muscle cells in IPAH identified enhanced activation of the hexosamine biosynthetic pathway, which resulted in increased glycosylation and proliferation of cells leading to typical morphologic hallmarks of PAH. Additionally, a partial knockdown of this metabolic pathway resulted in the reversal of cell proliferation. Interestingly, increased levels of glycosylation products in patients with IPAH were linked to an increased risk of hospitalization, lung transplantation, and mortality [32].

To the best of our knowledge, this is the first report to date from a large multicentre registry that included a selected population of patients with IPAH, which constitutes a major strength of this study. We analysed prospective data from a nationwide registry (BNP-PL), which allowed us to include a large homogenous group of patients with IPAH representing the whole Polish adult population diagnosed and treated for this rare disease [13]. The included individuals were diagnosed and followed by PH reference centres accredited by the Polish National Health Fund and dedicated to the treatment of patients with PAH and chronic thromboembolic pulmonary hypertension. In our study, the high number of WHO FC I or II we assign to the fact that a significant number of included patients were already treated with PAH-specific therapies when enrolled to BNP-PL database. This is in line with the previous characterization of our population of PAH patients [13, 23, 24, 33]. Thanks to the prospective design of the registry, we were able to assess follow-up data, including mortality, of the recruited patients. Finally, understanding the effect of DM on the prognosis of patients with IPAH is clinically significant since it has been proposed as a novel risk factor in this population. A limitation of our study is the definition of patients with DM. At the start of the study, the measurement of HbA_1c_ levels was not widely validated as a diagnostic tool for DM. Another limitation is that due to the multicentre design of the study, we were not able to analyse the clinical course of DM and its management during the follow-up period. We also acknowledge that the precise duration of DM prior to IPAH diagnosis could be valuable information for enhancing the quality of the manuscript. However, due to the design of the BNP-PL multicentre registry, this information was not initially collected during the data collection phase. Therefore, we were unable to include this information in the analysis. Further work is needed to understand the implications behind the relationship between the clinical course of DM and the outcomes of patients with IPAH. In our study, it is important to highlight that a significant proportion of the patients exhibited comorbidities such as hypertension, coronary artery disease, and renal failure, which are recognized risk factors for diastolic dysfunction of the left ventricle. To ensure the exclusion of patients with group 2 pulmonary hypertension from our study, we followed the ESC guidelines algorithm, which relied on measuring PAWP. Only patients with PAWP ≤ 15 mmHg were included in the present study. However, it is worth noting that mild forms of diastolic dysfunction of the left ventricle are common in PAH and do not rule out the diagnosis. A study conducted by Tonelli et al. reported that impaired relaxation of the left ventricle and pseudonormalization were observed in 88% and 2% of the patients, respectively [34]. Although we did not specifically assess diastolic left ventricular dysfunction in our study, it is crucial to consider the potential impact of such dysfunction on the results. It could act as a confounding factor when evaluating the relationship between diabetes mellitus and all-cause mortality.To address the potential confounding effect of diastolic dysfunction on the association between DM and all-cause mortality, further research is warranted. This would contribute to obtaining a more comprehensive understanding of the relationship between DM and mortality, taking into account the influence of diastolic dysfunction and other relevant factors. It is also important to acknowledge that approximately 20% of the study population had asthma or chronic obstructive pulmonary disease as comorbid conditions, which is consistent with data from other large PAH registries [35]. Exclusion of group 3 pulmonary hypertension was determined by the investigators at each pulmonary hypertension center and based on tests recommended by ESC guidelines, including pulmonary function tests, arterial blood gases, and chest high-resolution CT scans conducted during the diagnostic workup. Nevertheless, the presence of these comorbidities remains a potential confounding factor. Therefore, it is necessary to interpret the findings within the context of this potential confounding effect, as these respiratory conditions can have complex interactions with DM and independently impact patients’ outcomes.

## Conclusions

Our study demonstrated that patients with IPAH and DM present with more advanced pulmonary vascular disease and have worse survival than their counterparts without DM. Importantly, these findings remain significant even after accounting for age, BMI, and associated cardiovascular comorbidities.

## Data Availability

The datasets used and analysed during the current study are available from the corresponding author on reasonable request.

## Abbreviations

BMI: Body mass index
BNP‑PL: Database of Pulmonary Hypertension in the Polish population
CI: Cardiac index
DM: Diabetes mellitus
ESC: European Society of Cardiology
IPAH: Idiopathic pulmonary arterial hypertension
mPAP: Pulmonary artery pressure
PAH: Pulmonary arterial hypertension
PAWP: Pulmonary artery wedge pressure
PVR: Pulmonary vascular resistance
RAP: Right atrial pressure
6MWD: 6-minute walk distance
SvO_2_: Mixed venous oxygen saturation
WHO-FC: World Health Organization functional class
